# PLLA Composites Combined with Delivery System of Bioactive Agents for Anti-Inflammation and Re-Endothelialization

**DOI:** 10.3390/pharmaceutics14122661

**Published:** 2022-11-30

**Authors:** Seung-Woon Baek, Da-Seul Kim, Duck Hyun Song, Semi Lee, Jun-Kyu Lee, So-Yeon Park, Jun Hyuk Kim, Tae-Hyung Kim, Chun Gwon Park, Dong Keun Han

**Affiliations:** 1 Department of Biomedical Science, CHA University, 335 Pangyo-ro, Bundang-gu, Seongnam-si 13488, Republic of Korea; 2 Department of Biomedical Engineering, SKKU Institute for Convergence, Sungkyunkwan University (SKKU), 2066 Seobu-ro, Jangan-gu, Suwon-si 16419, Republic of Korea; 3 Department of Intelligent Precision Healthcare Convergence, SKKU Institute for Convergence, Sungkyunkwan University (SKKU), 2066 Seobu-ro, Jangan-gu, Suwon-si 16419, Republic of Korea; 4 School of Integrative Engineering, Chung-Ang University, 84 Heukseok-ro, Dongjak-gu, Seoul 06974, Republic of Korea; 5 Division of Biotechnology, College of Life Sciences and Biotechnology, Korea University, Seoul 02841, Republic of Korea

**Keywords:** vascular regeneration, angiogenesis, poly(L-lactide), magnesium hydroxide, polydeoxyribonucleotide, arginine, extracellular vesicle

## Abstract

The development of a biodegradable vascular scaffold (BVS) for the treatment of cardiovascular diseases (CVDs) still requires some improvement. Among them, re-endothelialization and anti-inflammation are clinically important to restore vascular function. In this study, we proposed a coating system to deliver hydrophilic bioactive agents to BVS using nanoemulsion and drop-casting methods. The poly(L-lactide) (PLLA) scaffold containing magnesium hydroxide (MH) was coated on the surface with bioactive molecules such as polydeoxyribonucleotide (PDRN), L-arginine (Arg, R), and mesenchymal stem cell-derived extracellular vesicles (EVs). PDRN upregulates the expression of VEGF as one of the A2A receptor agonists; and Arg, synthesized into nitric oxide by intracellular eNOS, induces endothelialization. In particular, EVs, which are composed of a lipid bilayer and transfer bioactive materials such as protein and nucleic acid, regulate homeostasis in blood vessels. Such a bioactive agent coating system and its PLLA composite suggest a new platform for the treatment of cardiovascular dysfunction.

## 1. Introduction

Although the development of a biodegradable vascular scaffold (BVS) for the treatment of atherosclerotic cardiovascular diseases has been recently highlighted, it still requires some improvement. Cardiovascular inflammation after BVS implantation occurs due to increased local acidity caused by acidic degradation products of poly(L-lactic acid) (PLLA), the main component of BVS, which has become a major factor inducing neointima formation and restenosis [1,2,3]. The promotion of re-endothelialization at the site of vascular injury is the most desirable factor for inhibiting neointimal formation [4]. There are various approaches to ameliorate the function of BVS with anti-inflammation and re-endothelialization, clinically important characteristics to restore vascular function after implantation. Among them, to improve these properties, many strategies have been devoted to developing a bioactive agent coating system for BVS. Zhang et al. reported enhanced endothelial cell function by constructing an epigallocatechin (EGCG)-coating system using the layer-by-layer (LBL) method [5]. Kersani et al. developed a drug-coating system with hydrophilic polymers on the vascular stent by the electrospinning method [6]. Abraham et al. coated on a stent using only proteins without polymers to improve anti-inflammation and anti-thrombosis [7]. However, these coating methods using hydrophilic materials would be possible to wash out by rapid blood flow during and after implantation into arteries. Therefore, to design a functionalized BVS, there is still a limitation in maintaining and releasing the desired active agents on BVS for a long time.

Generally, magnesium hydroxide [Mg(OH)_2_, MH], basic inorganic particles, can buffer acidic degradation products of biodegradable aliphatic polyesters such as PLLA, poly(ε-caprolactone) (PCL), and poly(L-lactide-co-glycolide) (PLGA) during the hydrolytic degradation process [8,9,10]. Therefore, inflammatory response by degradation products of BVS is inhibited by MH. In our prior research, the MH-incorporated PLLA scaffold maintained a neutral pH and decreased the expression of proinflammatory cytokines [11,12,13].

Polydeoxyribonucleotide (PDRN) is an oligo DNA extracted from salmon trout (*Oncorhynchus mykiss*) gonads and has an excellent ability for angiogenesis [14,15]. It acts as an A2A receptor agonist, upregulating the expression of angiogenic factors such as vascular endothelial growth factor (VEGF) [15]. Since it has a DNA structure, PDRN provides nucleotides and nucleosides as building blocks via the salvage pathway.

Nitric oxide (NO) is a signaling molecule with important biological and chemical functions in almost all physiological systems in the body [16,17,18,19]. NO promotes proliferation and migration of endothelial cells. Moreover, it plays an important role in maintaining blood vessels such as inhibiting proliferation and migration of smooth muscle cells and aggregation and activation of platelets [20]. In addition, arginine (Arg, R) is synthesized into NO by three types of nitric oxide synthase including endothelial nitric oxide synthase (eNOS), inducible nitric oxide synthase (iNOS), and neural nitric oxide synthase (nNOS) in endothelial cells [21,22,23].

Meanwhile, extracellular vesicles (EVs) are nanovesicles (50–150 nm) secreted by cells and act as signaling molecules containing various RNAs and proteins. For decades, stem cells have been clinically considered as a promising therapeutic cell source since they have various functions, including self-renewal abilities and differentiation. However, many recent studies have emphasized stem cell-derived EVs, which have a similar effect as MSC, including enhancing cell proliferation [24], anti-inflammation [25,26], angiogenesis [25,27,28], and cell differentiation [29].

**Scheme 1:**
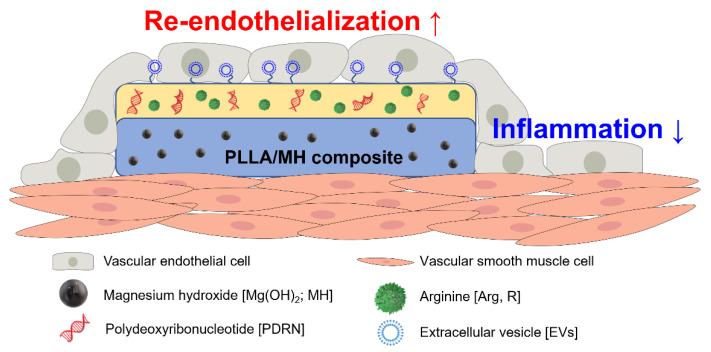
A schematic illustration of the PLLA composites including MH, PDRN, Arg, and MSC-derived EVs.

In this study, we proposed a sustained-release coating system to deliver these hydrophilic bioactive agents on BVS using nanoemulsion and drop-casting methods, and prepared the PLLA composites, including MH, PDRN, Arg, and MSC-derived EVs, using this system (Scheme 1). The inflammatory response, NO release, and re-endothelialization properties were investigated using human umbilical vein endothelial cells (HUVECs) in vitro. This proposed coating system to deliver bioactive agents is expected to endure a sustained release for a long time, controlled by the degradation rate of hydrophobic polymers. It suggests a new therapeutic approach for atherosclerotic cardiovascular diseases.

## 2. Materials and Methods

### 2.1. Materials

Poly(L-lactide) (PLLA, Purasorb^®^ PL 32, Mw 320 KDa) was purchased from Corbion (Amsterdam, Netherlands). Poly(D,L-lactide) (PDLLA, R205S, Mw 10–18 kDa) was acquired from Evonik Industries AG (Essen, Germany). Magnesium hydroxide [Mg(OH)_2_; MH], L-arginine (Arg, R), Tween^®^80, and polyethyleneimine (PEI) were purchased from Sigma-Aldrich (St. Louis, MO, USA). Polydeoxyribonucleotide (PDRN) was supplied from GoldBio (St. Louis, MO, USA). Chloroform (CF) and tetrahydrofuran (THF) were obtained from Daejung Co. Ltd. (Seoul, Republic of Korea). The protease K and Universal RNA Extraction Kit were purchased from Bioneer (Daejeon, Republic of Korea).

Umbilical cord mesenchymal stem cells (UC-MSCs) were purchased from CHA Biotech Co. Ltd. (Seongnam, Republic of Korea). Dulbecco’s Modified Eagle Medium (DMEM), fetal bovine serum (FBS), and phosphate-buffered saline (PBS) solution were purchased by Hyclone (Cytiva, MA, USA). Human umbilical cord-derived mesenchymal stem cells (UC-MSCs) were provided from CHA Biotech Co. Ltd. (Seongnam, Republic of Korea). The antibiotic antimycotic solution (A/A) was obtained from Gibco (Thermo Fisher Scientific, Waltham, MA, USA).

Human umbilical vein endothelial cells (HUVECs) and EGM-2 media bullet kit were purchased by Lonza (Basel, Switzerland). Cell-count kit (CCK-8) was obtained from Dongin LS (Hwaseong, Republic of Korea). The Live/Dead staining kit (calcein AM/EthD-1), nuclease-free water, and lipophilic tracer DiO were obtained from Invitrogen (Thermo Fisher Scientific, Waltham, MA, USA). A 4-amino-5-methylamine-2′,7′-difluorofluorescein diacetate (DAF-FM diacetate) was purchased from Cayman Chemical (Ann Arbor, MI, USA). Radioimmunoprecipitation assay (RIPA) Buffer 1× (9806S) was obtained from Cell Signaling (Danvers, MA, USA). PrimeScript RT Reagent Kit (Perfect Real Time) was purchased from Takara (Tokyo, Japan).

### 2.2. Preparation and Characterization of the PLLA Composites

The MH-incorporated PLLA composite (PM) was fabricated by the solvent-casting method and thermally processed using a hot press, as the previous study [11,12,13]. The PLLA composites with PDRN and arginine (PMP and PMPR) were prepared by drop-casting technique. PDLLA was dissolved in tetrahydrofuran and chloroform (7:3) with 2 wt% Span 80. Totals of 10 mg PDRN and 15 mg arginine were dissolved in 60 μL nuclease-free water. Subsequently, this solution was dispersed in 1 mL PDLLA solution. The 100 μL dispersion was drop-casted on PM (20 × 20 mm) and then the solvent was evaporated. To immobilize the extracellular vesicles (EVs), the surface of the PMPR was coated with PEI. The PMPR was immersed in PEI solution (1 mg/mL in nuclease-free water) at room temperature overnight. Continuously, the PMPR-PEI was immersed in 500 μL EV solution (1 × 10^9^ particles/mL in PBS solution) at 37 °C overnight. The surface morphologies and immobilized EVs of the PLLA composites were observed by field-emission scanning electron microscopy (FE–SEM; S–4800, Hitachi, Tokyo, Japan) at 15 kV with SE mode. The loading amount of MH was measured using thermogravimetric analysis (TGA; TGA 4000, PerkinElmer, Waltham, USA). To measure the loading amounts of PDRN and Arg, the surface of the composites was dissolved in tetrahydrofuran. The solvent was evaporated and then 1 mL of water was added. PDRN and Arg concentrations were detected by Nanodrop and UV/vis (200 nm) (ND-1000; Thermo Fisher Scientific, Waltham, MA, USA), respectively. To demonstrate the immobilized EVs on the surface, the composites were incubated at 2.5 μg/mL with 500 μL lipophilic tracer DiO at 37 °C for 30 min, and then estimated using confocal laser scanning microscopy (Zeiss LSM880; Carl Zeiss, Oberkochen, Germany).

### 2.3. MSC-Derived EV Isolation

UC-MSCs were cultured up to almost 50% confluence in alpha-MEM (HyClone, Cytiva, MA, USA) containing 1% A/A and 10% FBS and in a humidified atmosphere with 5% CO_2_ at 37 °C. Cell culture medium was changed and collected every 12 h with DMEM (without phenol red) containing 1% A/A and EV-depleted FBS using ultracentrifugation. Obtained media was centrifuged at 1300 rpm for 3 min and filtered by 0.22 um filter. For EV isolation, a tangential flow filtration (TFF; Repligen, Waltham, MA, USA) system with a 500 kDa fiber membrane was used as previously reported [30,31]. The collected EVs were centrifuged at 6000× *g* for 30 min and filtered through a 0.22 um filter.

### 2.4. Characterization of MSC-Derived EVs

The morphology of EVs was confirmed using transmission electron microscopy. The size and concentration of the particle were determined using ZetaView QUATT^®^ (Particle Metrix, Meerbusch, Germany). To demonstrate the characteristic of EVs, the same number of EVs (1.78 × 10^8^ particles) were analyzed by Western blot. EVs were incubated with RIPA buffer for 30 min and centrifuged for 10 min at 4 °C. The proteins of EVs were separated on 10% SDS-PAGE and transferred to nitrocellulose (NC) membranes. The membranes were blocked for 1 h at room temperature using 5% skim milk dissolved in Tris-buffered saline with Tween 80 (TBS-T) solution. The primary antibody of each membrane was labeled with anti-CD81 (1:200; Santa Cruz Biotechnology, Dallas, TX, USA), anti-CD63 (1:500; Abcam, Cambridge, UK), anti-CD9 (1:200; Santa Cruz Biotechnology, Dallas, TX, USA), and anti-Alix antibody (1:200; Santa Cruz Biotechnology, Dallas, TX, USA).

### 2.5. Dispersion Stability Test

The dispersion turbidity was investigated using a SpectraMax M2 Microplate Reader (Molecular Device; Sunnyvale, CA, USA). The PDLLA solution containing PDRN and Arg with or without Span 80 was measured at a wavelength of 550 nm for 1 h.

### 2.6. Degradation and Release Behavior

To evaluate the degradation behavior of the samples (20 × 10 mm), the pH and mass changes were investigated in 1 mL PBS solution with 20 μg/mL proteinase K at 37 °C for 7 days. The proteinase K was added every two days. The released MH amount was measured by inductively coupled plasma–optical emission spectroscopy (ICP-OES, Optima 8000, PerkinElmer, Waltham, MA, USA). The PDRN release was measured in PBS solution at 37 °C for 1 month. The PDRN concentration was measured by Nanodrop (ND-1000; Thermo Fisher Scientific, Waltham, MA, USA). The MSC-derived EVs release was observed in PBS solution at 37 °C for 48 h. The MSC-derived EVs concentration was also measured by Nanodrop.

### 2.7. Cell Culture and Cell Viability Assay

HUVECs were cultured in EGM-2 media with 5% CO_2_ at 37 °C. The cells were seeded into a 24-well culture plate at the density of 1 × 10^4^ cells/well and the PLLA composites (10 × 10 mm) were placed in each well using 24-well inserts. After 24 h, the cell viability was measured using CCK-8 assay and a Live-Dead kit following the manufacturer’s instructions.

### 2.8. RNA Extraction and Quantitative Real-Time PCR (qRT-PCR)

RNA was extracted using a Universal RNA Extraction Kit following the manufacturer’s protocol. Extracted RNA was reverse-transcribed to complementary DNA using a PrimeScript RT Reagent Kit following the manufacturer’s instruction. The qRT-PCR was performed using each primer and SYBR Green PCR Master Mix (Applied Biosystems, Thermo Fisher Scientific, Waltham, MA, USA). The expressions of proinflammation- and nitric oxide-related genes were calculated with the 18S rRNA as a reference gene using the 2^−∆∆Ct^ method. Forward and reverse primer sequences were designed for IL-6, IL-8, eNOS, HIF-1α, CAT1 α, ANG1, VEGF, and 18S rRNA as follows: IL-6 (forward: 5′-gatgagtacaaaagtcctgatcca-3′, reverse: 5′-ggcagccactggttctgt-3′), IL-8 (forward: 5′-agacagcagagcacacaagc-3′, reverse: 5′-atggttccttccggtggt-3′), eNOS (forward: 5′-atccagtgccctgcttca-3′, reverse: 5′-gcagggcaagttaggatcag-3′), HIF-1α (forward: 5′-tttttcaagcagtaggaatt-3′, reverse: 5′-gtgatgtagtagctgcatga-3′), CAT1α (forward: 5′-cacagtggccaggatccaat-3′, reverse: 5′-ctgcaaacaccagccagttc-3′), ANG1 (forward: 5′-tccacataggaaatgaaaagca-3′, reverse: 5′-cagcaccgtgtaagatcagg-3′), VEGF (forward: 5′-actggaccctggctttactg-3′, reverse: 5′-tctgctccccttctgtcgt-3′), and 18S (forward: 5′-cctggataccgcagctagga-3′; reverse: 5′-gcggcgcaatacgaatgcccc-3′).

### 2.9. DAF-FM Analysis

To investigate the NO release in vitro, HUVECs (1 × 10^4^ cells/well) were seeded into a 24-well plate and incubated for 1 day. The PLLA composites were placed in each well using 24-well inserts and incubated. After 1 day, each well was treated with 5 μmol DAF-FM and reacted at 37 °C for 30 min. The released NO was observed using fluorescence microscopy (U-RFL-T, Olympus, Tokyo, Japan). The fluorescence intensity was analyzed by Image J software (National Institutes of Health, Bethesda, MD, USA).

### 2.10. Wound-Healing Assay/Migration Assay

To assess of wound closure effect of the PLLA composites, the cell monolayer scratching method was used. HUVECs (1.5 × 10^5^ cells/well) were seeded into a 6-well plate and incubated for 1 day. Cells grown as a confluent monolayer were scratched using a 1 mL pipette tip and washed with PBS solution. The PLLA composites were cocultured with the inserts and the wound area was observed after 12 h using optical microscopy (CKX53, Olympus, Japan). Healing of the wound area was quantified by Image J software (National Institutes of Health, Bethesda, MD, USA).

### 2.11. Tube Formation Assay

To evaluate the angiogenesis effect of the PLLA composites, 300 uL of the Matrigel matrix (356234, Corning) was coated on the 24-well plates at 37 °C incubator for 1 h. HUVECs (1.2 × 10^5^ cells/well) were seeded and the PLLA composites were cocultured with Transwell inserts. After 16 h, inserts were removed and cells were stained with calcein AM (4 μmol) following the manufacturer’s instructions. The calcein AM-stained cells were observed using a fluorescence microscopy. Quantification of the branch point and tube length of each group were analyzed by Image J software.

### 2.12. Statistical Analysis

All quantitative results were obtained through more than three independent results, and the values were expressed as mean ± standard deviation (SD). The statistical significance was compared by one-way ANOVA using Tukey’s post hoc method in Graph-Pad Prism 7.0 software (GraphPad Software, Inc., San Diego, CA, USA). * *p* < 0.05, ** *p* < 0.01, *** *p* < 0.001, and # *p* < 0.0001 indicated statistically significant difference, respectively.

## 3. Results and Discussion

### 3.1. Preparation and Characterization of The PLLA Composites

The MH-incorporated PLLA composite (PM) was prepared with solvent-casting and hot-pressing methods based on our previous studies [11,12]. Then, the surface of PM was coated using a biodegradable polymer solution in which PDRN and Arg were dispersed. The polymer solution with PDRN and Arg was prepared by the nanoemulsion method using Span 80. As a final process, its surface was coated with PEI, then MSC-derived EVs were immobilized on its surface.

**Figure 1:**
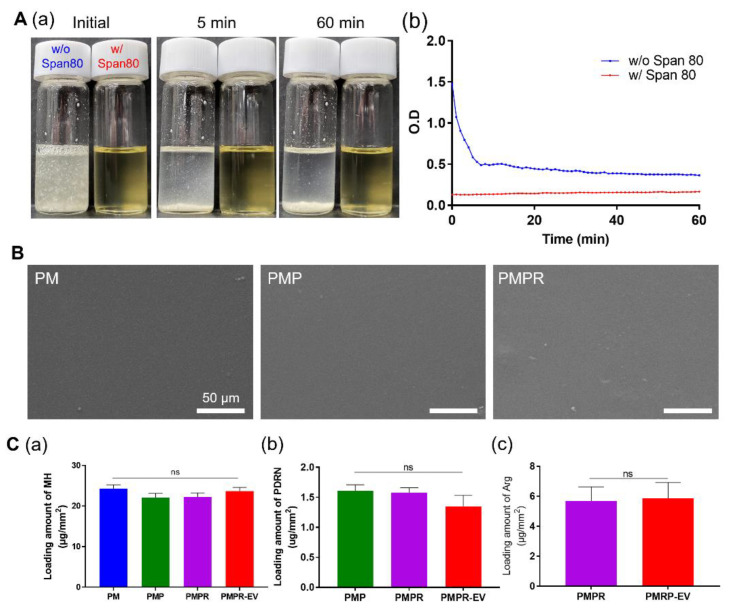
Characterization of the PLLA composites. (**A**) Dispersion stability of the coating solution; dispersion images (**a**) and turbidity assay (**b**) at 550 nm for 1 h. (**B**) The representative SEM images of the PM, PMP, and PMPR. (**C**) Quantification of the loading amount of MH (**a**), PDRN (**b**), and Arg (**c**) in each composite.

PDRN and Arg are aggregated and precipitated in organic solvents due to their hydrophilic properties. To disperse them in a polymer solution, a water solution incorporating bioactive molecules was emulsified with Span 80 on organic solvent. The Span 80, an oil-soluble surfactant, has a low hydrophile–lipophile balance (HLB) value of 4.3, which is more stable at forming a water-in-oil (w/o) emulsion [32]. Figure 1A displays that the addition of Span 80 enhanced the dispersion stability of the coating solution. PDRN and Arg were aggregated and precipitated as soon as they were added to the solution without Span 80 (w/o Span 80).

The turbidity assay showed initial high absorbance by the aggregated bioactive agents without Span 80 (w/o Span 80). Afterward, the absorbance decreased due to the precipitation of the molecules. On the other hand, the absorbance of the sample with Span 80 (w/ Span 80) remained in a clear and transparent state and continuously exhibited a low absorbance, even after 1 h.

Figure 1B shows SEM images of the surface of the PM, PM coated with PDRN (PMP), and PM coated with PDRN and Arg (PMPR). All composites appeared as smooth surfaces and there was no significant difference between composites. To measure the distribution of the bioactive agents in the composites, the composites were stained with DAPI which could label PDRN, and observed using fluorescence microscopy (Figure S1). The composite without PDRN showed no fluorescence, whereas the composite with PDRN exhibited widely distributed fluorescence without any aggregation. Figure 1C shows the loading amount of each component. The loading amounts of MH, PDRN, and Arg were analyzed by TGA (Figure S2), nanodrop, and UV/Vis spectrophotometer, respectively. About 23 μg/mm^2^ of MH, 1.51 μg/mm^2^ of PDRN, and 5.76 ug/mm^2^ of Arg were incorporated in the PMPR. To immobilize the EVs, the surface of the PMPR was coated with PEI [33]. The PEI contains many amine functional groups, so it can induce ionic bonds between negatively charged polymers and EVs. The surface charge of the PMPR, which was −45 mV, increased to −3 after PEI coating (Figure S3). In addition, the water contact angles of the PM, PMP, and PMPR were 73.67, 79.16, and 80.08, respectively (Table S1), while the water contact angle of PMPR-PEI and PMPR-EV decreased to 56.59 and 20.18, respectively, indicating that the surface of the hydrophobic composite was coated with hydrophilic PEI. These results demonstrated that the PEI was successfully coated on the surface of the PMPR.

**Figure 2:**
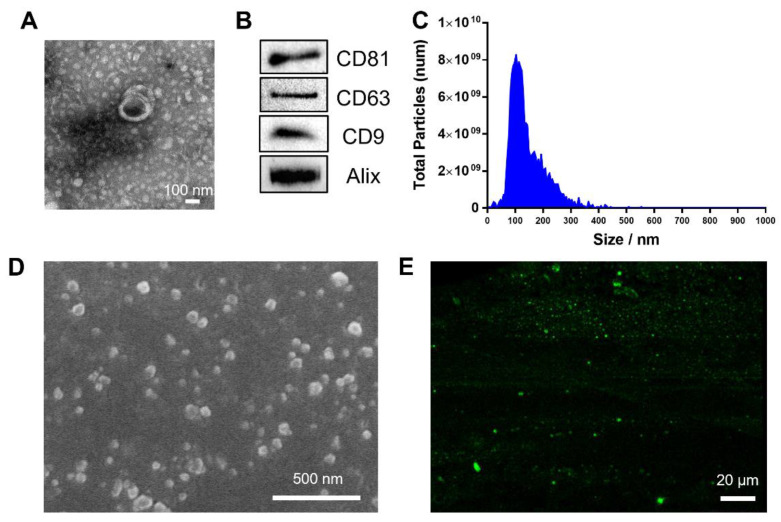
Characterization of MSC-derived EVs. (**A**) Representative TEM image of isolated EVs. (**B**) Western blot analysis showing the expression of specific markers in EVs. (**C**) The size distributions of EVs using Zetaview. (**D**) The representative SEM and (**E**) confocal scanning microscopy images of immobilized EVs on the surface of PMPR-EV.

Figure 2 shows the characterization and immobilization on the surface of the composites of MSC-derived EVs. The phospholipid bilayer structure and the approximately 204 nm diameter of MSC-derived EVs were observed through the TEM image (Figure 2A). Figure 2B shows that the isolated EVs had typical transmembrane markers of EVs, such as through Western blot analysis. The sizes of MSC-derived EVs were further analyzed using Zetaview. They ranged from 50 to 300 nm and had an average size of 169 nm. The particle numbers were observed as 1.73 × 10^11^ (Figure 2C). Isolated MSC-derived EVs were immobilized on the surface of PMPR-PEI (PMPR-EV), and then the distribution of immobilized EVs by SEM and confocal scanning microscopic images were displayed. SEM images showed that they had the spherical shape of typical EVs and were distributed on the surface of the composite, whereas they was not observed in the composite without EV immobilization (Figure 2D and Figure S4). To accurately confirm the distribution of MSC-derived EVs over a wider area on the composites, DiO staining, which can specify fluorescent stains with a lipid layer of EVs, was performed (Figure 2E and Figure S5). After immobilization of MSC-derived EVs in the PMPR and PMPR-PEI, they were stained with DiO (green). Fluorescent signals could not be observed on the surface of PMPR, whereas were evenly distributed over a large area on the PMPR-PEI.

### 3.2. Degradation and Release Behavior

**Figure 3:**
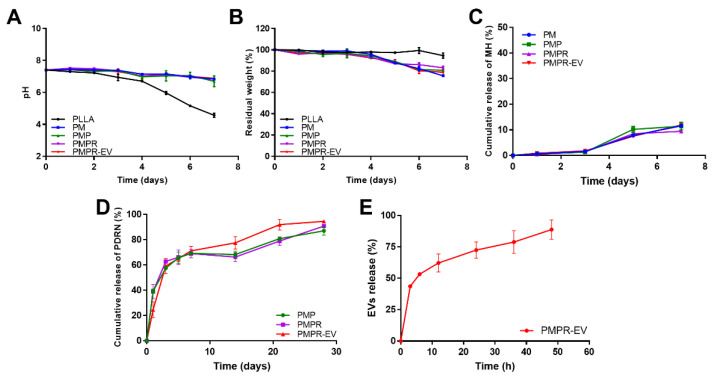
Degradation and release behavior of the composites. (**A**) The pH value, (**B**) residual weight, and (**C**) cumulative release of MH of the composites during accelerated degradation in PBS solution with protease K at 37 °C for 7 days. Cumulative release of (**D**) PDRN and (**E**) EVs in the composites for 28 days.

The degradation behavior of the composites and MH release were conducted under accelerated conditions for 7 days with proteinase K using a known method in many other studies [11,12,15,34,35]. In Figure 3A, the pH value of the PLLA started to gradually decrease after 3 days and drastically decreased to 4.57 after 7 days. Bartkowiak-Jowsa et al. reported no change in the pH value of PLLA in a ten-month degradation period [36]. It can be inferred that 3 days under our accelerated condition is more than ten months in the non-accelerated condition. In contrast, the pH value of the PM, PMP, PMPR, and PMPR-EV slightly decreased to 6.84, 6.70, 6.83, and 6.88, respectively, because of the pH neutralization ability of MH. The residual weight of PLLA decreased to 94.5% at 7 days. However, the residual weight of the PM, PMP, PMPR, and PMPR-EV decreased to 75.65%, 80.77%, 83.14%, and 78.92%, respectively (Figure 3B). Their rates of mass loss were accelerated by multiple effects such as the release of MH, water penetration accelerated by MH, and degradation of PLLA by hydroxy groups in MH. These results could reduce the side effects of long-term implantation by degrading the implanted BVS faster than the degradation period of 2 years [37,38]. Figure 3C shows the MH release of the PM, PMP, PMPR, and PMPR-EV during degradation. All composites released about 10.96% of MH for 7 days. The PM was prepared using high pressure and heat, so that the MH was strongly surrounded by the PLLA. It is expected that most MH will remain to neutralize the acidic byproducts until PLLA is completely degraded through the comparison of accelerated and non-accelerated conditions mentioned above. The release behaviors of PDRN and EVs were investigated at 37 °C for 28 days. PDRN in the PMP, PMPR, and PMPR-EV was initially burst-released at 65.66%, 66.16%, and 65.04%, respectively, and then gradually released by 87.02%, 90.77%, and 94.52% until 28 days, respectively (Figure 3D). EVs released from PMPR-EV were measured for 48 h and released up to 88.78%. EV release tended to be the opposite tendency of MH release, because EVs were immobilized on the surface of the PMPR by an ionic bond using PEI. Despite the different release rates, our coating system exhibited a longer release time compared to other systems that showed a release curve of 6 h by coating bioactive agents on hydrophilic polymers [6].

### 3.3. Biocompatibility of The PLLA Composites

**Figure 4:**
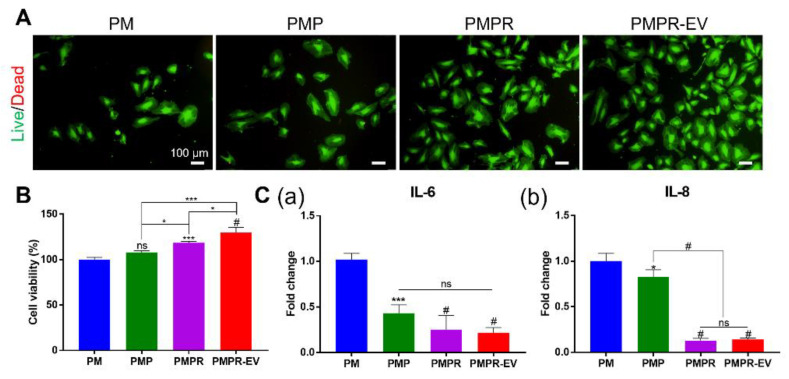
(**A**) Live-Dead assay images and (**B**) cell viability of HUVECs onto the composites at 24 h. (**C**) Gene expressions of IL-6 (**a**) and IL-8 (**b**) for the anti-inflammatory effect in HUVECs onto the composites. * *p* < 0.05, *** *p* < 0.001, and # *p* < 0.0001 indicate statistically significant differences, respectively.

To investigate the biocompatibility of the composites in vitro, a Live-Dead assay (Figure 4A) and cell viability test (Figure 4B) were performed with HUVECs. Since the MH incorporated-PLLA composite already showed good biocompatibility in our previous research [11,12], there were rare dead cell signals in all groups. With the addition of PDRN, Arg, and EVs on the composites, the proportion of calcein AM-positive cells (live cells) gradually increased. Especially, the PMPR-EV group observed a significantly large number of live cells. Moreover, the PMPR-EV enhanced the cell proliferation of HUVECs at 24 h. Other studies have noted that umbilical cord serum-derived exosomes enhance the proliferation, migration, and tube formation of HUVECs. Likewise, EV extracted from UC-MSC seems to have similar effects [39]. In addition, the anti-inflammation effect of the composites was evaluated (Figure 4C). Representative inflammatory cytokines, interleukin-6 (IL-6) and interleukin–8 (IL-8), were analyzed at the gene level. Gene expression levels of both cytokines significantly decreased in the PMP, PMPR, and PMPR-EV. Consequently, PMPR-EV was no cytotoxicity to cells, enhances cell proliferation, and had anti-inflammatory effects.

### 3.4. Confirmation of NO Releasing Ability and Effects of NO

**Figure 5:**
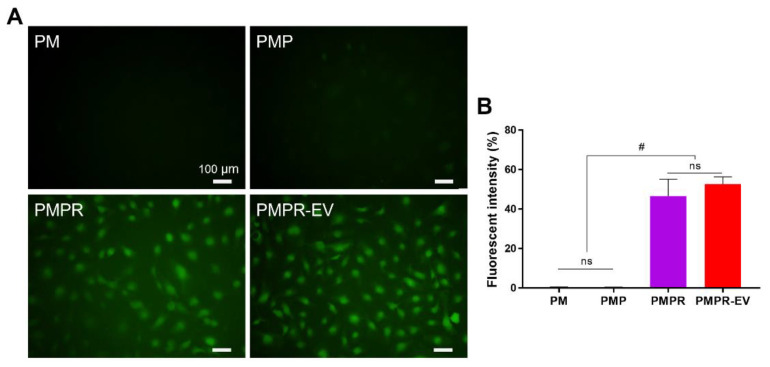
NO release from HUVEC onto the composites in vitro. DAF-FM stained (**A**) fluorescence images and (**B**) quantification of relative fluorescence intensity. # *p* < 0.0001 indicates statistically significant differences.

The NO release from HUVECs on the composites was observed using DAF-FM staining. DAF-FM is the most commonly used NO fluorescent probe in vitro. It reacts with NO to produce 4-amino-5-methylamino-2′,7′-difluorofluorescein triazole (DAF-FM-T), which emits fluorescence [40,41]. Figure 5A displays fluorescence images and Figure 5B shows the quantification of relative intensity by fluorescence images of each composite. The PMPR and PMPR-EV exhibited relatively higher intensity than PM and PMP because the production of NO increased by Arg. The NO generation by Arg existed not only inside the cells but also in media, and generally, fluorescence was observed.

### 3.5. Various Effects of the Composites on HUVECs

**Figure 6:**
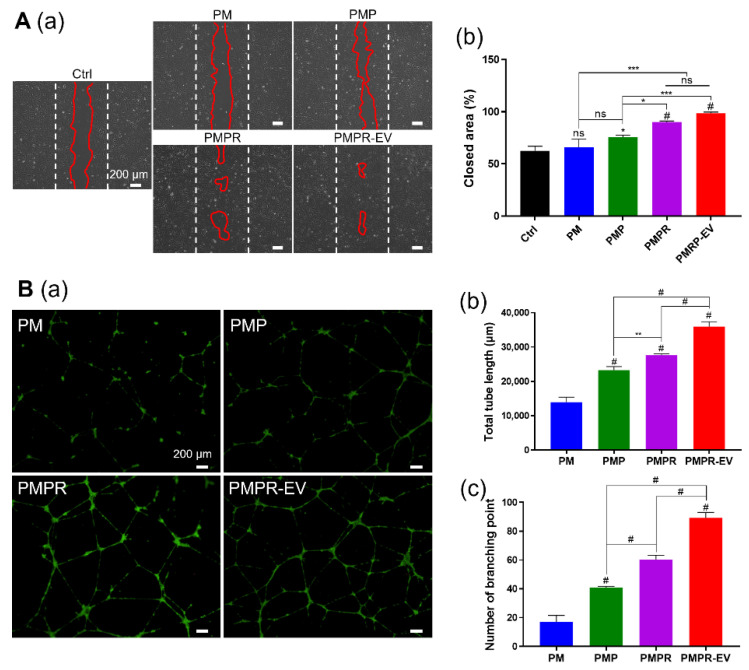
Re-endothelialization effects of the composites. (**A**) Optical images (**a**) and the ratio of the closed area (**b**) of wound-healing assay at 12 h. (**B**) Fluorescence images stained with calcein AM (**a**), quantification of total tube length (**b**), and the number of branch points (**c**) of the tubule-forming assay. * *p* < 0.05, ** *p* < 0.01, *** *p* < 0.001, and # *p* < 0.0001 indicate statistically significant differences, respectively.

To investigate the mitogenic capacity of the composites, the scratch assay was executed. The migratory ability of HUVECs is a critical factor that can promote re-endothelialization of implanted BVS, reduce thrombosis, and inhibit neointima formation and restenosis [42]. Figure 6A shows the optical images and ratio of the closed area at 12 h after scratching the plate and treating the composites. The closed area of the PM was similar to that of the control group. In contrast, the closed area of the PMP, PMPR, and PMPR-EV significantly increased to 75.86%, 90.24%, and 98.58%, respectively, because PDRN, Arg, and EVs could enhance growth and migratory ability. In Figure 6B, the proangiogenic ability of each composite was demonstrated through tube formation assay. These results observed that total tube length and the number of branch points significantly increase in the order of PM, PMP, PMPR, and PMPR-EV by the synergy effect of PDRN, Arg, and MSC-derived EVs. To conclude, the improved migratory and tube-forming ability of the developed composites is expected to enable rapid re-endothelialization after BVS implantation, and to resist thrombosis, neointima formation, and restenosis.

### 3.6. Genetic Assessment to Verify the Angiogenic Effect

**Figure 7:**
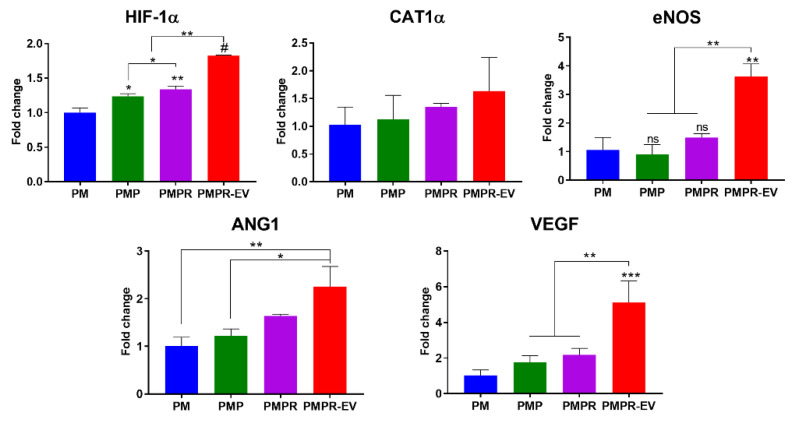
Genetic evaluation of HUVECs using NO-related genes, including eNOS, HIF-1α, CAT1α, ANG1, and VEGF by qRT-PCR. * *p* < 0.05, ** *p* < 0.01, *** *p* < 0.001, and # *p* < 0.0001 indicate statistically significant differences, respectively.

NO is synthesized by nitric oxide synthase (NOS), which oxidizes guanidine nitrogen of Arg in endothelial cells. In normoxia conditions, NO stimulates various inter- and intracellular signalings, including vasodilation, cell migration, and angiogenesis. Additionally, EVs are also well-known to have proangiogenesis-promoting effects, like the cell, MSC, from which it originated [43]. To evaluate the angiogenic effect of the composites, a genetic assessment was conducted using angiogenesis-associated markers by qRT-PCR (Figure 7).

Hypoxia-inducible factor-1α (HIF-1α) is a pivotal regulator of angiogenesis. HIF-1α stimulates the induction of iNOS in various cell types, constituting a positive feedback mechanism between NO production and HIF-1α stabilization [44]. NO-mediated induction of hypoxia contributes to increased HIF-1α expression levels. Moreover, Zhang et al. investigated that MSC-derived EVs promoted HIF-1α expression resulting in the proliferation, migration, and tube formation of endothelial cells [45]. The mRNA expression level of HIF-1α was notably enhanced in the PMPR-EV compared to that of the PM (*p* < 0.0001). Cationic amino acid transporter 1α (CAT1α) is the principal Arg transporter expressed in endothelial cells, which is colocalized with eNOS in the plasma membrane. Although there were no statistical differences between the composites, the CAT1α expressions slightly increased as bioactive molecules were added. Among NOS signaling pathways, it was hypothesized that the eNOS-dependent pathway plays the major role in NO production. The expression of eNOS of the PMPR-EV increased by about 3.42-, 3.98-, and 2.44-fold than PM, PMP, and PMPR, respectively.

Consequently, angiopoietin 1 (ANG1) and VEGF gene expressions of the PMPR-EV were significantly upregulated on HUVECs. These results revealed that NO-induced local hypoxia condition could cause vasodilation of epithelial cells and promote the infiltration of PDRN and EVs.

## 4. Conclusions

The developed coating system for the delivery of hydrophilic bioactive agents showed high coating stability and sustained release, and the prepared PLLA composites using this coating system observed anti-inflammation and re-endothelialization effects in vitro. In particular, the PMPR-EV showed related improvement in endothelial cell proliferation, NO production, and angiogenic gene expressions. Taken together, the synergistic interaction of PDRN, Arg, and EVs alleviated inflammation and facilitated angiogenesis. When this coating system is applied to BVS, it is expected that it is possible for it to address and overcome side effects such as inflammation, thrombosis, neointima formation, and restenosis and become a new treatment technology for cardiovascular disease.

In the future study, we plan to devise a BVS with this coating system and evaluate its inhibitory effects on thrombosis, neointima formation, and restenosis.

## Data Availability

The data presented in this study are available on request from the corresponding author.

## Supplementary Materials

The following supporting information can be downloaded at: https://www.mdpi.com/article/10.3390/pharmaceutics14122661/s1, Table S1: Water contact angle (WCA) on the surface of the composites; Figure S1: To display the distribution of PDRN, fluorescence images were stained with DAPI of PM and PMP; Figure S2: TGA thermograms of the PLLA, MH, PM, PMP, PMPR, PMPR-PEI, and PMPR-EV; Figure S3: Surface charges of PMPR and PMPR-PEI; Figure S4: SEM images of PMPR without EV immobilization; Figure S5: Confocal laser scanning microscopy image of PMPR after incubation of EV solution.
