# Supplementary material for: PLLA Composites Combined with Delivery System of Bioactive Agents for Anti-Inflammation and Re-Endothelialization
Source: Pharmaceutics. 2022 Nov 30;14(12):2661. doi: 10.3390/pharmaceutics14122661 (PMC9782680; doi:10.3390/pharmaceutics14122661)
Supplement: Supplementary file 1 [file pharmaceutics-14-02661-s001.zip › pharmaceutics-2040802-supplementary.pdf]

Supplementary Information

PLLA Composites Combined with Delivery System of Bioactive Agents for Anti-Inflammation and Re-Endothelialization

Seung-Woon Baek [1,2,3,†], Da-Seul Kim [1,4,†], Duck Hyun Song [1], Semi Lee [1], Jun-Kyu Lee [1], So-Yeon Park [1,5], Jun Hyuk Kim [1], Tae-Hyung Kim [4], Chun Gwon Park [2,3] and Dong Keun Han [1,*]

[1] Department of Biomedical Science, CHA University, 335 Pangyo-ro, Bundang-gu, Seongnam-si 13488, Republic of Korea
[2] Department of Biomedical Engineering, SKKU Institute for Convergence, Sungkyunkwan University (SKKU), 2066 Seobu-ro, Jangan-gu, Suwon-si 16419, Republic of Korea
[3] Department of Intelligent Precision Healthcare Convergence, SKKU Institute for Convergence, Sungkyunkwan University (SKKU), 2066 Seobu-ro, Jangan-gu, Suwon-si 16419, Republic of Korea
[4] School of Integrative Engineering, Chung-Ang University, 84 Heukseok-ro, Dongjak-gu, Seoul 06974, Republic of Korea
[5] Division of Biotechnology, College of Life Sciences and Biotechnology, Korea University, Seoul 02841, Republic of Korea
* Correspondence: dkhan@cha.ac.kr
† These authors contributed equally to this work.

Table S1. Water contact angle (WCA) on the surface of the composites

Sample	WCA (degree)
PM	73.67±0.52
PMP	79.16±2.01
PMPR	80.08±5.71
PMPR-PEI	56.59±0.77
PMPR-EV	20.18±4.81

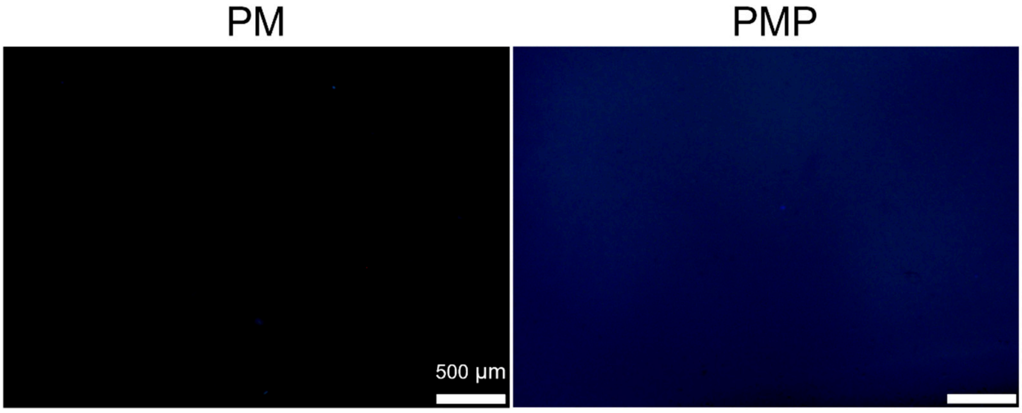

Figure S1. To display the distribution of PDRN, fluorescence images were stained with DAPI of the PM and PMP.

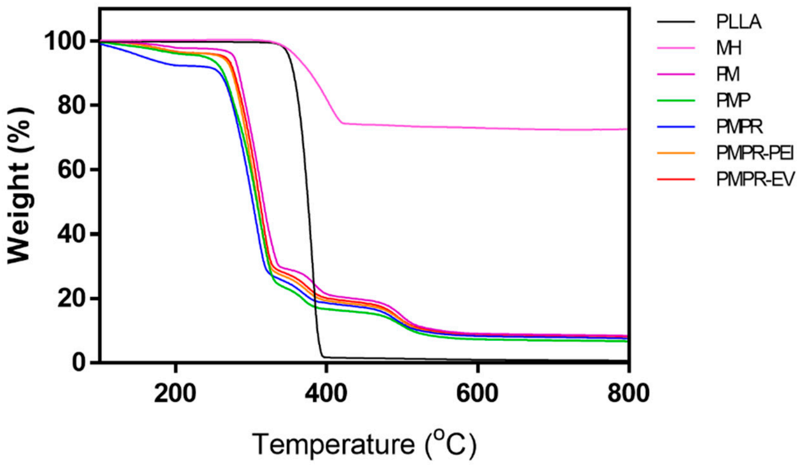

Figure S2. TGA thermograms of the PLLA, MH, PM, PMP, PMPR, PMPR-PEI, and PMPR-EV.

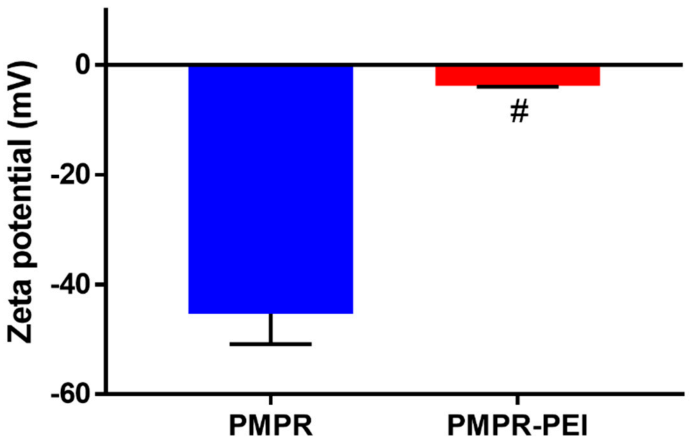

Figure S3. The surface charges of PMPR and PMPR-PEI. #$p < 0.0001$ indicate statistically significant differences.

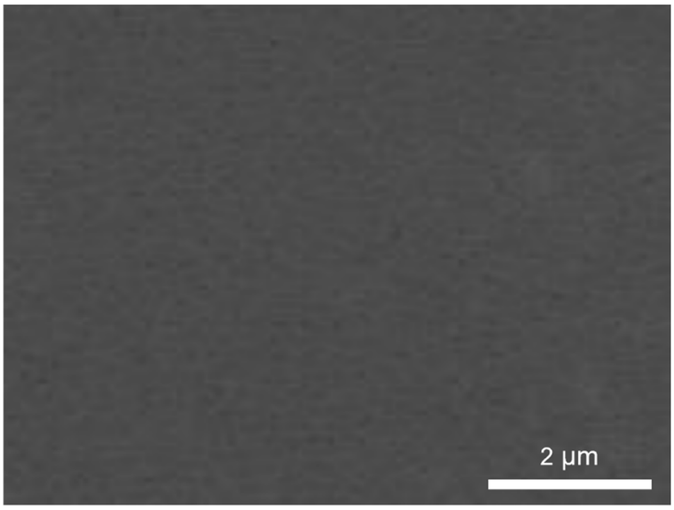

Figure S4. SEM images of PMPR without EVs immobilization.

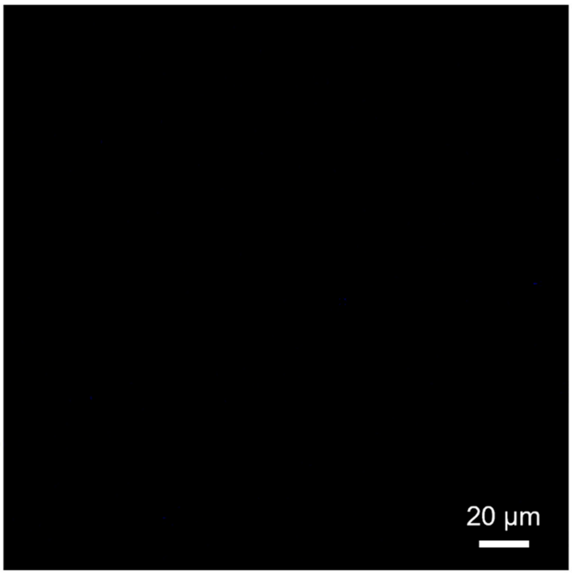

Figure S5. Confocal laser scanning microscopy image of PMPR after incubation of EVs solution.
